# Exosomal lncRNA LINC01356 Derived From Brain Metastatic Nonsmall-Cell Lung Cancer Cells Remodels the Blood–Brain Barrier

**DOI:** 10.3389/fonc.2022.825899

**Published:** 2022-04-27

**Authors:** Sumin Geng, Shaohua Tu, Zhenwei Bai, Yixiong Geng

**Affiliations:** ^1^ Department of Neurosurgery, Beijing Tiantan Hospital, Capital Medical University, Beijing, China; ^2^ China National Clinical Research Center for Neurological Diseases, Beijing, China; ^3^ School of Basic Medical Sciences, Capital Medical University, Beijing, China

**Keywords:** lung cancer, lncRNA LINC01356, exosome, BBB, brain metastasis

## Abstract

Brain metastasis is a severe complication that affects the survival of lung cancer patients. However, the mechanism of brain metastasis in lung cancer remains unclear. In this study, we constructed an *in vitro* BBB model and found that cells from the high-metastatic nonsmall cell lung cancer (NSCLC) cell line H1299 showed a higher capacity to pass through the blood–brain barrier (BBB), as verified by Transwell assays, than cells from the low-metastatic NSCLC cell line A549. Brain microvascular endothelial cells (BMECs) could internalize H1299-derived exosomes, which remarkably promoted A549 cells across the BBB. The BBB-associated exosomal long noncoding RNA (lncRNA) was selected from the RNA-Seq dataset (GSE126548) and verified by real-time PCR and Transwell assays. LncRNA LINC01356 was significantly upregulated in H1299 cells and exosomes derived from these cells compared to that of A549 cells. Moreover, LINC01356 was also upregulated in serum exosomes of patients with NSCLC with brain metastasis compared with those without metastasis. In addition, BMECs treated with LINC01356-deprived exosomes expressed higher junction proteins than those treated with the control exosomes, and silencing LINC01356 in exosomes derived from H1299 cells could inhibit A549 cells from crossing the BBB. These data might indicate that the exosomal lncRNA LINC01356 derived from brain metastatic NSCLC cells plays a key role in remodeling the BBB system, thereby participating in brain metastasis in lung cancer.

## Introduction

Patients with lung cancer initially present with brain metastases in 10%–25% of cases, with up to 50% of patients developing brain metastases throughout their disease course (1). Survival of patients with brain metastasis (BM) is limited to mere weeks, extended to months upon administration of multidisciplinary treatment (2–5). The blood–brain barrier (BBB) plays an important role in BM because one of the vital steps in the complex process of BM is the migration of metastatic cells through the BBB. The BBB regulates homeostasis of the central nervous system by forming a tightly regulated neurovascular unit that includes endothelial cells (ECs), pericytes and astrocytes, which together maintain normal brain function (6, 7). However, it is still largely unknown how cancer cells pass through the BBB to cause BM.

Long noncoding RNAs (lncRNAs) are transcripts longer than 200 nucleotides with no protein-coding capacity that drive many important cancer phenotypes through their interactions with other cellular macromolecules, including DNA, protein, and RNA (8). Some lncRNAs play critical roles in regulating the BBB and blood-tumor barrier (BTB) permeability in some brain tumors (9, 10). LncRNAs are also important during the metastasis of cancer (11–14). Extracellular vesicles, a heterogeneous group of cell-derived membranous structures comprising exosomes (30–100 nm in diameter) and microvesicles, which are continuously secreted by cells to the extracellular environment, represent a novel vehicle for cell-cell communication and are involved in multiple physiological and pathological processes (15, 16). Exosomes, of endocytic origin and that are released into the extracellular space by all cell types through the fusion of multivesicular bodies, carry proteins, RNAs (mRNAs, non­coding RNAs including microRNAs) and DNA sequences, for example, tumor­associated molecules in the case of cancer and premetastatic niche establishment (15–21). A variety of molecules carried within exosomes, such as miRNAs and lncRNAs, can be transferred from exosome-producer and recipient cells and are involved in the tumor microenvironment and metastasis (22–29). However, it remains poorly understood whether and how lung cancer cell-secreted exosomal lncRNAs regulate the BBB and are involved in brain metastases.

In this study, we investigated the roles of exosomal lncRNAs in the brain metastasis of NSCLC by using the highly metastatic NSCLC cell line H1299, the low-metastatic NSCLC cell line A549 and an *in vitro* model of BBB.

## Materials and Methods

### Cell Culture

H1299 cells were cultured in RPMI 1640 medium (10-040-CVR, CORNING) supplemented with 10% heat-inactivated FBS (Invitrogen) and antibiotic–antimycotic agents at 37°C in 5% CO^2^ RPMI-1640. A549 cells were cultured in DMEM/F12 medium (10-092-CVR, CORNING) supplemented with 10% heat-inactivated FBS (Invitrogen) and antibiotic–antimycotic agents at 37°C in 5% CO^2,^ and human brain vascular pericytes (HBVPs) and brain capillary epithelial cells (BMECs, Procell) were cultured in ECM medium (sciencell 1001) containing 10% FBS at 37°C in an atmosphere of 5% CO^2^.

### Transwell Assay

Transwell assays were used to determine migration and invasive ability. Cell migration was assessed using 0.8 μm 24-well chambers (353097, Falcon), and cell invasion was assessed using BioCoat™ Matrigel^®^ 0.8 μm 24-well chambers (354480, BioCoat). Cells 1×10^5^ were plated in the top chamber. The lower chamber contained 700 μl of medium (DMEM/F12 CORNING; RPMI 1640 (CORNING) supplemented with 10% FBS (21-040-CV, CORNING). The cells were incubated for 24 h, cells in the upper chamber were removed, and cells on the lower surface were stained with 0.1 crystal violet (C0121 blue sky).

### BBB *In Vitro* Model

To investigate the function of exosomes in the BBB, we established an *in vitro* BBB model as previously described (25). HBVP was plated on the lower surface of the upper chamber, and BMECs were plated in the upper chamber. Astrocytes were cultured in the lower chamber. The barrier function of the BBB model was evaluated by determining its transepithelial/transendothelial electrical resistance (TEER).

### Exosome Isolation

Cancer cells were cultured, and the medium was then replaced with serum-free medium and cultured for another 48 hours. The culture was centrifuged at 300 g for 10 min. The supernatant was collected, and dead cells were removed by centrifugation at 2000×g for 10 min, followed by centrifugation at 10000×g for 30 min to remove cell debris. The supernatant was ultracentrifuged at 4°C and 100000×g for 1 h. This step was repeated. The supernatant was removed, and the exosome precipitate was added to precooled 1× phosphate-buffered saline (PBS). The exosomes were verified by transmission electron microscopy (TEM), the exosomes diameter was measured by Nanoparticle Tracking Analysis (NTA). The protein expression is verified by SDS page analysis and CD63 protein was verified through western blotting (WB).

### Exosome Tracing Experiment

Exosomes were extracted from 1.5x10^6^ cells, then the exosomes were labeled with DiI dye (Blue sky#C1036) and incubated for 20 min. The cells were incubated with mouse brain microvascular endothelial cells, and exosomes were located by observing fluorescence.

### Transwell Invasion Assay Using the *In Vitro* BBB Model

Cancer cell invasion was assessed in an *in vitro* BBB model. The cells were trypsinized and labeled with green fluorescent protein (GFP). Cancer cells were plated at a density of 2 × 10^4^ cells in serum-free DMEM, and RAPM-1640 medium containing 10% serum was used as the chemoattractant in the lower chamber. After 48 h, the noninvading cells were removed, and the invading cells were labeled with GFP. All assays were performed in triplicate.

### Screening for Brain Metastasis-Related lncRNAs

To explore which exosomal lnc-RNAs are involved in lung cancer BM, we used primary lung tumor RNA-Seq data (GSE126548) from patients with BM and non-metastatic NSCLC for analysis. First, we downloaded GSE126548 data, using the DeSeq package analysis of BM and transfer of NSCLC patients differentially expressed lncRNA, significance of difference threshold is set to FDR < 0.05, | log2 fold change | > 1. Then, analyzed the target lncRNA differential expression by miRanda (http://www.microrna.org/miRanda) and RNAhybrid (https://bibiserv.cebitec.uni-bielefeld.de/rnahybrid/). The function of target Genes was predicted by Gene Ontology (GO), the Kyoto Encyclopedia of Genes and Genomes (KEGG) analyzed the signaling pathways involved in target Genes.

### Real-Time PCR

The expression of lncRNAs was validated by real-time PCR. Total RNA from the exosomes and cells was extracted by TRIzol reagent (Invitrogen). The quantity and quality of the extracted RNA was determined by a NanoDrop 2000 spectrophotometer (Wilmington, DE, USA). Qualified RNA samples with an A260/280 ratio >1:9 were used for real-time PCR. A PrimeScript RT kit (Takara Bio, Dalian, China) was used to synthesize complementary DNA (cDNA). Real-time PCR was performed using a SYBR-Green PCR kit (Roche Diagnostics, Indianapolis, IN, USA). PCR was performed on an ABI QuantStudio™6 Flex System. U6 was used as the internal reference, and the primer sequences used for PCR are shown in **Table 1**. The PCR was run at 95°C for 10 min for denaturation, 45 cycles of 95°C for 15 sec, and 60°C for 60 sec and dissociation at 95°C for 10 sec, 60°C for 1 min, and 95°C for 15 sec. The data were analyzed by the 2 −ΔΔCt method. The PCRs were all repeated three times.

**Table 1:** Primer sequence.

Gene symbol	Primer sequence
SLC7A11-AS1	F 5’ ACCAGACAGCAGTGCTCAAG 3’ R 5’ GAGAACTGACAGCCAGGGAA 3’
LINC01356	F 5’ CTTTCCACGCGCTTGTTTCG 3’ R 5’ CCCAAGACGTAGAGCTTCCC 3’
LINC01252	F 5’ AAAAGACGGCCTCTGTCTGG 3’ R 5’ TGTCCTGACACTTCTGCCAC 3’
CTD-2319I12.2	F 5’ GGCTTGGAGATTTTTCGCCG 3’ R 5’ AGCCTTCCTCCTCCTGTGAT 3’
CTA-373H7.7	F 5’ CACCCGAGGTCCAAAGAGAA 3’ R 5’ ATGAGTCCCCTCAGATGCAG 3’

### Western Blot

Proteins from exosomes and BMECs were extracted, and the concentration was determined using a BCA assay kit (Pierce Biotechnology, Inc., Rockford, IL, USA). The resolved proteins were transferred to polyvinylidene fluoride membranes (Millipore, Bedford, MA, USA). The proteins were blocked in 5% milk and incubated with primary antibodies against CD63 (1:1000, EXOAB-CD63A-1, SBI), ZO-1 (1:1000, 13663S, Cell Signaling Technology), claudin-5 (1:1000, Ab131259, Abcam), N-cadherin (1:5000, Ab76011, Abcam), Occludin (1:1000, Ab216327, Abcam) and glyceraldehyde-3-phosphate dehydrogenase (GAPDH, 60004-1-Lg, Proteintech) overnight at 4°C. The samples were then incubated with anti-rabbit or anti-mouse horseradish peroxidase-conjugated secondary antibodies. Enhanced chemiluminescence reagent (Thermo Fisher Scientific, Waltham, MA, USA) was used to show the protein bands, and optical density was assessed *via* ImageJ software.

### Statistical Analysis

GraphPad Prism 8.0 and SPSS 25 were used to analyze the real-time PCR data. Two-tailed Student’s t-test was used to compare the differences between two groups. The mean value ± standard deviation was used to present the experimental data. A P < 0.05 was considered statistically significant. Cell number was counted by ImageJ software.

## Results

### Confirmation of Brain Metastatic Cell Lines

**Figure 1:**
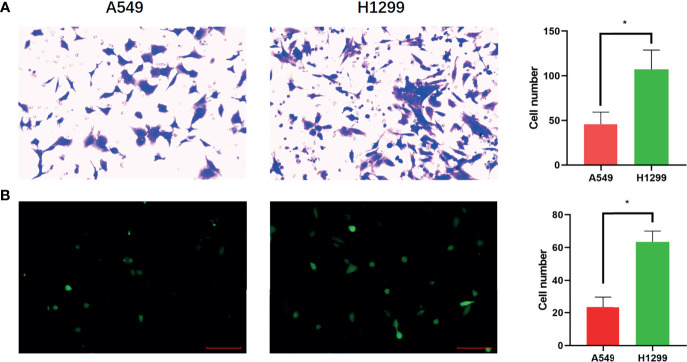
Confirmation of brain metastatic cell lines. **(A)** Transwell assay shows the migration ability of A549 and H1299 cells and cell numbers of A549 and H1299 cells in the Transwell assay. **(B)** Transwell assay shows the invasive ability of A549 and H1299 cells and cell numbers of A549 and H1299. (N = 3, one-way ANOVA, *P < 0.05).

A previous study showed that H1299 is a highly invasive cell line and that A549 cells are minimally invasive (28). Transwell assays were used to confirm the invasion of H1299 and A549 cells. The results demonstrated that both the migration and invasion abilities of H1299 cells were significantly higher than those of A549 cells (**Figure 1**).

### Establishment of an *In Vitro* BBB Model

**Figure 2:**
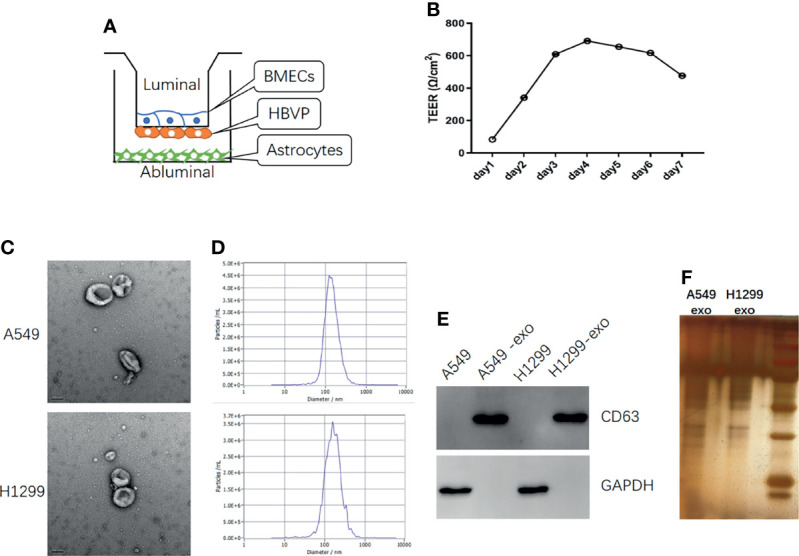
The *in vitro* BBB model establish and identification of exosomes derived from NSCLC cells. **(A)** Schematic diagram of the *in vitro* BBB model, *in vitro* **(B)** The curve over time of TEER established in the *in vitro* BBB model. **(C)** TEM identification of exosomes from A549 and H1299 cells. **(D)** The NTA analysis of the diameter of exosomes from A549 and H1299 cells. **(E)** Western blot identification of the exosome marker CD63 in cells and exosomes. **(F)** SDS page analysis of exosomes protein expression.

To better investigate the invasion of lung cancer cells into the brain, we established an *in vitro* BBB model. The *in vivo* BBB commonly consists of BMECs, pericytes, and astrocytes. Therefore, we seeded BMECs on the upper part of the upper compartment. Then, the pericytes were seeded on the lower part of the upper compartment, and the astrocytes were seeded on the lower compartment (**Figure 2A**). The conjunction of the *in vitro* model was confirmed by TEER, and the TEER increased in 3-4 days and remained at a sufficiently high level (**Figure 2B**).

### Identification of Exosomes Derived From NSCLC Cells

To explore the influence of exosomes on the BBB *in vitro* model, we extracted exosomes from H1299 and A549 cells, which were identified through TEM. (**Figure 2C**) and the signature protein CD63 (**Figure 2E**). The exosomes diameter was analyzed by NTA (**Figure 2D**) and the protein expression was identified by SDS page analysis (**Figure 2F**). TEM revealed that the extracted particles were 100 to 150 nm in size with a complete membrane structure, which corresponded with exosomes, while WB results showed that these particles significantly expressed the exosome marker CD63. NTA revealed that the peak diameter of particles from A549 is 128.1nm and the full width at half maxima (FWHM) is 119.3nm, while the peak diameter of particles from H1299 is 158.6nm and the FWHM is 82.4nm, which indicate that the main component of particles is exosomes.

### Exosomes From H1299 Cells Can Promote Cancer Cell Invasion in an *In Vitro* BBB Model

**Figure 3:**
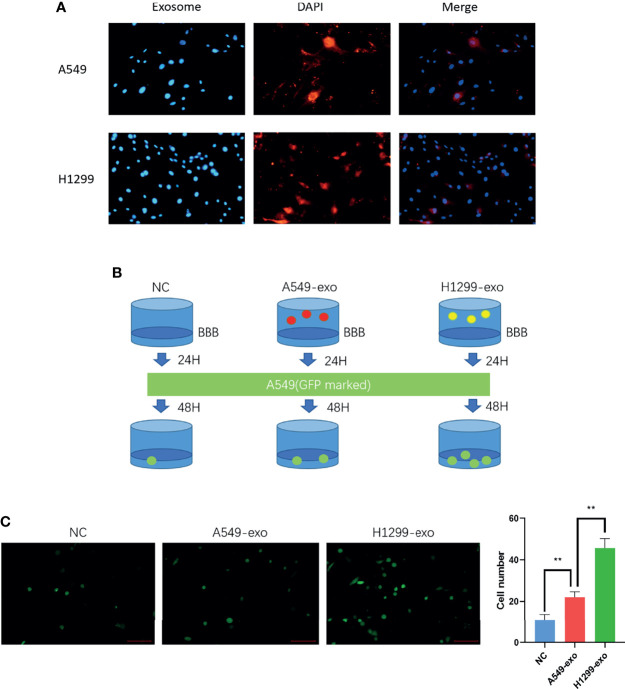
The exosomes from a highly metastatic cell line can promote cancer cell invasion in an *in vitro* BBB model. **(A)** DiI dye tracing of exosomes from A549 and H1299 cells. BMECs internalized exosomes after 24 incubation. **(B)** Schematic diagram of the Transwell assay for cancer cell-derived exosomes promoting A549 cells to penetrate the BBB model. The *in vitro* BBB model was incubated with exosomes for 24 h, and then GFP-marked A549 cells were cultured for another 48 h. **(C)** Exosomes were added to the BBB *in vitro* model and incubated for 24 hours. Then, A549 cells were added, and the number of cells crossing was counted after 48 hours (magnification ×200, one-way ANOVA, *P < 0.05, **P < 0.01).

The exosomes were labeled with DiI dye added to the *in vitro* BBB model cocultivation for 24 hours separately. The figures show that the exosomes from H1299 and A549 cells could be absorbed by BMECs (**Figure 3A**). Then, to determine whether NSCLC with low invasiveness could penetrate the BBB model treated with exosomes, we counted the A549 cells that were processed by H1299 and A549 exosomes and crossed the BBB, which were labeled with GFP (**Figure 3B**). The results demonstrated that A549 cells penetrated the *in vitro* BBB model treated with H1299 exosomes more than those treated with A549 exosomes (**Figure 3C**).

### Exosomes Derived From NSCLC Cells Remodel the BBB by Carrying LINC01356

**Figure 4:**
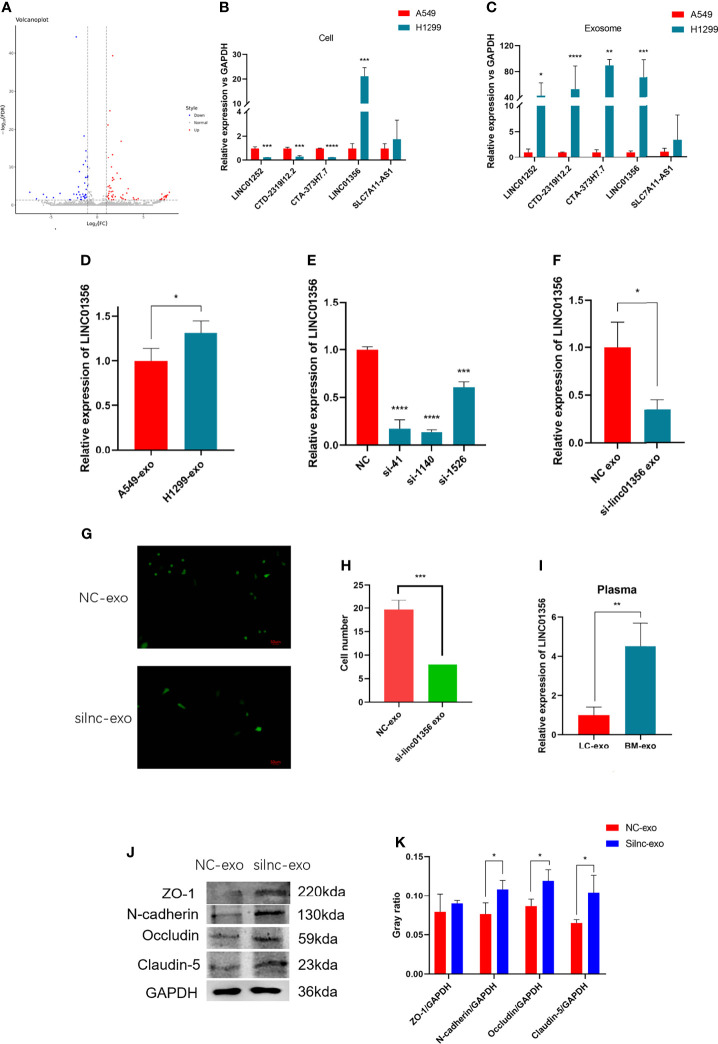
Mechanism of exosomal LINC01356 *in vitro* BBB model remodeling and lung cancer brain metastasis. **(A)** Volcano plot of differentially expressed lncRNAs between brain metastatic and nonbrain metastatic lung cancer cells. Data were obtained from the GSE126548 database. **(B)** The top five most highly differentially expressed lncRNAs were verified by RT–PCR in A549 and H1299 cells. **(C)** The top five most highly differentially expressed lncRNAs were verified by RT–PCR in exosomes from A549 and H1299 cells. **(D)** LINC01356 expression level of BMECs incubated with exosomes from A549 and H1299 cells. **(E)** Different interference rates of siRNAs were verified by RT–PCR. **(F)** LINC01356 expression level of BMECs incubated with NC-Exos and silnc-Exos. **(G)** Immunofluorescence image and number of A549 cells crossing through the BBB *in vitro* model treated with NC-Exos and silnc-Exos. **(H)** Cell number of Fig. 3g. **(I)** Expression level of LINC01356 in LC-Exos and BM-Exos. **(J)** Western blotting was used to detect the expression of tight junction and adhesive proteins in BMECs treated with NC-Exos and silnc-Exos. **(K)** Gray ratio of western blot for junction protein. The expression levels of ZO-1, N-cadherin, Occludin and Claudin-5 protein increased when siRNA interfered with LINC01356. Among them, the expression of ZO-1 was not statistically significant. (NC-Exo= exosomes derived from H1299 cells without siRNA transfection, silnc-Exo= exosomes derived from H1299 cells treated with siRNA, LC-Exo= plasma exosomes from lung cancer patients without metastasis, BM-Exo= plasma exosomes from lung cancer patients with brain metastasis, *P < 0.05, **P < 0.01, ***P < 0.001, ****P < 0.0001).

To further explore how H1299-derived exosomes promote A549 cells to cross the *in vitro* BBB model. We first analyzed the lncRNAs differentially expressed between H1299 and A549 cells from the RNA-Seq dataset GSE126548. A total of 99 differentially expressed lncRNAs were found. Compared with the exosomes of A549 low metastatic NSCLC cells, 59 lncRNAs were upregulated and 40 lncRNAs were downregulated in the exosomes of H1299 high metastatic NSCLC cells (**Figure 4A**). We screened five candidate lncRNAs, LINC01252, CTD-2319I12.2, CTA-373H7.7, LINC01356, and SLC7A11-AS1, which were stable and significantly increased in expression. Through qRT–PCR verification, we found that LINC01356 was significantly upregulated in both cells (**Figure 4B**) and exosomes (**Figure 4C**) in H1299 cells, and the expression level was the highest. Therefore, we further explored the effect of LINC01356 on the BBB.

First, we added exosomes from A549 and H1299 cells to BBB models *in vitro* and measured the expression level of LINC01356 in BMECs separately. The figure shows that LINC01356 is more highly expressed in exo-H1299-treated BMECs (**Figure 4D**). Then, we transfected small interfering RNAs (siRNAs) to interfere with the efficiency of LINC01356 siRNA silencing. Moreover, siRNA-1140 showed the highest interference effect and was selected for further investigation (**Figure 4E**). siRNA-1140 was then used to transfect H1299 cells to generate LINC01356-deficient silnc-Exos, and LINC01356 expression was dramatically decreased in silnc-Exos (**Figure 4F**). Finally, we compared the invasive ability of A549 cells treated with normal H1299 exosomes (NC-Exos) and LINC01356-deficient exosomes (silnc-Exos) through Transwell assays. The results showed that A549 cells treated with NC-Exos successfully crossed the *in vitro* BBB model. However, the infiltrated cell number of A549 cells treated with silnc-Exos was lower. (**Figures 4G, H**). To further study the effect of LINC01356 on cell junctions, we detected the expression of tight junction proteins in BMECs. The expression levels of ZO-1, Occludin, Claudin and N-cadherin proteins were significantly decreased in the BMECs transfected with NC exosomes compared to those transfected with siRNA exosomes (**Figures 4J, K**).

### Exosomal lncRNA LINC01356 Expression Is Higher in the Serum of Patients With Brain Metastases

To further confirm whether the expression level of LINC01356 is higher in patients with brain metastases, we collected six lung cancer patient plasma samples with (n=3) or without (n=3) brain metastasis. The level of LINC01356 in exosomes was determined by qRT–PCR. The level of exosomal LINC01356 was significantly higher in the plasma of patients with brain metastasis (**Figure 4I**).

## Discussion

The BM of lung cancer is an important factor affecting patient survival. The mechanism of tumor metastasis to the brain is currently unclear. Fortunately, emerging evidence suggests that EVs (species of exosomes and shed microvesicles) have crucial roles in cancer development, including premetastatic niche formation and metastasis (25, 30, 31). For exosomal miRNAs derived from brain metastatic breast cancer cells, exosome-mediated transfer of cancer-secreted miR-105 destroys tight junctions and the integrity of the BBB (24), and cancer-derived EVs containing miR-181c trigger the breakdown of the BBB (25). For lncRNAs, exosomes derived from highly brain metastatic breast cancer cells might destroy the BBB system and promote the passage of cancer cells across the BBB by transferring lncRNA GS1-600G8.5 (29). These studies all indicate that EVs containing miRNAs and lncRNAs destroy the BBB and promote brain metastasis in breast cancer. However, there is a lack of relevant research on the mechanism of lung cancer brain metastasis. In this study, we first indicate that exosomal lncRNA LINC01356 derived from brain metastatic NSCLC cells remodels the BBB.

First, we compared the invasive ability between A549 and H1299 cells, and the results showed that the highly metastatic cell lineH1299 could infiltrate the *in vitro* BBB model more easily. To explore the mechanism, we investigated whether exosomes of A549 and H1299 cells play a role in this process, and the results revealed that H1299-derived exosomes can be absorbed by BMECs in an *in vitro* BBB model and improve the permeability of A549 cells, which indicated a crucial role of tumor-derived exosomes in BBB remodeling. To further explore the mechanism by which exosomes affect the *in vitro* BBB model. We analyzed the lncRNAs differentially expressed between H1299 and A549 cells in the data and validated them through qRT-PCR. The results indicated that exosome-derived LINC01356 is more highly expressed in both exosomes and H1299 cells, which may indicate that LINC01356 plays a key role in *in vitro* BBB model remodeling. Silencing LINC01356 remarkably inhibited the invasion ability of a lung cancer cell line in an *in vitro* BBB model. Finally, western blot analysis suggested that LINC01356 can affect the expression of the junction proteins Occludin, Claudin and N-cadherin. This evidence may indicate that exosomal LINC01356 can remodel the BBB in an *in vitro* model by decreasing the expression of Claudin, Occludin and N-cadherin. Most importantly, we found that plasma-derived exosomes from lung cancer patients with brain metastasis expressed significantly higher levels of LINC01356 than those from patients without brain metastasis. This finding may suggest that LINC01356 could also affect the BBB *in vivo* in lung cancer patients, and further research is needed to verify this notion.

Although exosomal LINC01356 proved to be a critical factor in lung cancer brain metastasis, the mechanism remains to be further explored. In addition, LINC01356 was higher in the plasma of lung cancer patients with brain metastasis, which means that LINC01356 could be a predictive factor of the likelihood of brain metastasis. Moreover, whether blocking exosomal LINC01356 can prevent brain metastasis in patients with lung cancer still needs to be further explored. Exosome-derived LINC01356 can change the permeability of the *in vitro* BBB model, and exosomes can carry molecules and drugs, which means that they can be used to promote the effect of drugs in the central nervous system. Therefore, exosomal LINC01356 may be a suitable candidate vehicle for drug delivery. We speculate that it may strengthen the therapeutic effect of drugs in primary central nervous system tumors or infections. Whether exosomal LINC01356 can be used in the adjuvant treatment of central nervous system diseases needs to be further explored. In conclusion, exosomal LINC01356 could be a novel predictive factor and therapeutic target for brain metastasis, and it may also be an adjuvant to drug therapy for central nervous system diseases.

## Conclusion

In conclusion, the high-metastatic NSCLC cell line H1299 could infiltrate the *in vitro* BBB model more easily than cells from the low-metastatic NSCLC cell line A549, and H1299-derived exosomes can be absorbed by BMECs in an *in vitro* BBB model and improve the permeability of A549 cells, which indicated a crucial role of tumor-derived exosomes in BBB remodeling. Furthermore, LncRNA LINC01356 was significantly upregulated in H1299 cells and exosomes derived from these cells compared to that of A549 cells, BMECs treated with LINC01356-deprived exosomes expressed higher junction proteins than those treated with the control exosomes, and silencing LINC01356 in exosomes derived from H1299 cells could inhibit A549 cells from crossing the BBB, which may indicate that exosomal LINC01356 can remodel the BBB in an *in vitro* model. Meanwhile, exosomal LINC01356 is more highly expressed in the plasma of NSCLC patients with brain metastasis. It may promote the process of lung cancer brain metastasis through remodeled BBB by targeting cell junction proteins.

## Data Availability Statement

The original contributions presented in the study are included in the article/supplementary materials. Further inquiries can be directed to the corresponding author.

## Author Contributions

SG: Experiment design and manuscript revision. ST: Writing manuscript. ZB: Experiment and data acquisition. YG: Experiment and data acquisition. All authors contributed to the article and approved the submitted version.

## Conflict of Interest

The authors declare that the research was conducted in the absence of any commercial or financial relationships that could be construed as a potential conflict of interest.

## Publisher’s Note

All claims expressed in this article are solely those of the authors and do not necessarily represent those of their affiliated organizations, or those of the publisher, the editors and the reviewers. Any product that may be evaluated in this article, or claim that may be made by its manufacturer, is not guaranteed or endorsed by the publisher.
